# Changes in the Potential Multiple Cropping System in Response to Climate Change in China from 1960–2010

**DOI:** 10.1371/journal.pone.0080990

**Published:** 2013-12-03

**Authors:** Luo Liu, Xinliang Xu, Dafang Zhuang, Xi Chen, Shuang Li

**Affiliations:** 1 State Key Laboratory of Desert and Oasis Ecology, Xinjiang Institute of Ecology and Geography, Chinese Academy of Sciences, Urumqi, China; 2 State Key Laboratory of Resources and Environmental Information Systems, Institute of Geographical Sciences and Natural Resources Research, Chinese Academy of Sciences, Beijing, China; 3 University of Chinese Academy of Sciences, Beijing, China; National Rice Research Center, United States of America

## Abstract

The multiple cropping practice is essential to agriculture because it has been shown to significantly increase the grain yield and promote agricultural economic development. In this study, potential multiple cropping systems in China are calculated based on meteorological observation data by using the Agricultural Ecology Zone (AEZ) model. Following this, the changes in the potential cropping systems in response to climate change between the 1960s and the 2010s were subsequently analyzed. The results indicate that the changes of potential multiple cropping systems show tremendous heterogeneity in respect to the spatial pattern in China. A key finding is that the magnitude of change of the potential cropping systems showed a pattern of increase both from northern China to southern China and from western China to eastern China. Furthermore, the area found to be suitable only for single cropping decreased, while the area suitable for triple cropping increased significantly from the 1960s to the 2000s. During the studied period, the potential multiple cropping index (PMCI) gap between rain-fed and irrigated scenarios increased from 18% to 24%, which indicated noticeable growth of water supply limitations under the rain-fed scenario. The most significant finding of this research was that from the 1960s to the 2000s climate change had led to a significant increase of PMCI by 13% under irrigated scenario and 7% under rain-fed scenario across the whole of China. Furthermore, the growth of the annual mean temperature is identified as the main reason underlying the increase of PMCI. It has also been noticed that across China the changes of potential multiple cropping systems under climate change were different from region to region.

## Introduction

Multiple cropping is a general cropping practice which involves growing two or more crops on the same field in a given year. It is one of the most effective ways to increase grain yield and to promote agricultural economic development. There is a common belief that food production and food security are continuously threatened by the increasing price of crude oil, extreme weather, and the diversion of crop land for bio-fuel production. Therefore, food security has long been an issue which has attracted global attention under a situation of urbanization by which the total area of active farmland continues to decrease. Insufficient attention has been paid to processes that increase crop yield, leading to an increase in the multiple cropping index (MCI; ratio of sown area to cropland area) [1]–[2]. Due to environmental limitations, increased food production is unlikely to be achieved from farmland expansion in many regions [3]. In this situation, multiple cropping is an effective method for improving crop productivity as it is an efficient use of natural resources (i.e. land, light, heat and so on) and human resources. This practice also helps to alleviate competition for land use between food production and economic crops.

China is one of the largest countries in the world and has succeeded in achieving the highest MCI. Nearly half of the cultivated land in China is subject to multiple cropping practices. This has resulted in a 25% increase in crop yields in the past few decades, which has enabled China to feed 22% of the world's population using only 7% of the world's cultivated land [4]. Thus, an increase in the use of multiple cropping practices has assisted China in successfully addressing the issue of food security. Two factors which further underline the importance of multiple cropping practices are the global trends of climate change and urbanization. Both of these factors have caused cropping systems to undergo significant change during the last several decades [5]–[10]. In the case of China, this has resulted in a decrease in cropland area by 15% [11] (primarily distributed in developed areas).

The statistical method used for acquiring information on cropping system changes was -based on administrative boundaries from the government, which ignored the spatial heterogeneity within the same administrative area and thus failed to represent the spatial characteristics of the multiple cropping systems [12]–[14]. These systems can be measured according to the phenological and ecological characteristics of crop growth. This is done by identifying the periods of cropping cycles from time series data collected by remote sensing systems including AVHRR/NDVI, SPOT VEGETATION and MODIS [15]–[18]. This method is restrained by the temporal resolution of remote sensing data, and therefore cannot capture the changes in the multiple cropping systems over long periods of time. However it is suitable for food security assessment and agricultural development planning, which aims to quantify long term sequenced MCI changes and investigate its response to global climate change.

In this study, the potential multiple cropping systems in China were calculated using the method of AEZ based on meteorological observation data. The spatio-temporal change patterns between 1960 and -2010 were also evaluated, via the spatial analysis techniques of the Geographic Information System (GIS). Finally, the characteristic variations of the potential cropping systems were analyzed, and their responses to climate change in different regions were illustrated.

## Data and Methods

### Data sources

The input data for this study included terrain elevation, soil, land use and meteorological data (including the monthly maximum air temperature, minimum air temperature, precipitation, relative humidity, wind speed and sunshine hours).

#### Terrain Elevation Data

The terrain elevation dataset derived from the Shuttle Radar Topography Mission C-band (SRTM) was the first publicly available near-global, high resolution raster Digital Elevation Model (DEM) [19]. The SRTM data was distributed with a 90 m spatial resolution around the Earth, reduced from the original 30 m resolution via averaging and sub-sampling. Numerous worldwide applied studies have used SRTM data for environmental analysis [20]–[21]. The SRTM Version 2 data (by naming convention SRTM3 for 3 arc sec data) was sourced from the Jet Propulsion Laboratory (http://srtm.csi.cgiar.org/SELECTION/inputCoord.asp).

#### Soil Data

Soil quality was determined by several parameters, including soil type, effective soil depth and soil water holding capacity. A nation-wide soil dataset at the scale of 1∶1,000,000 was provided by the Data Center for Resources and Environmental Sciences at the Chinese Academy of Sciences (RESDC) (http://www.resdc.cn/first.asp). Soil data was used to calculate the soil-water balance, which determined the potential and actual evapotranspiration for a reference crop and the length of its growing period (LGP, days).

#### Land Use Data

A land-use database with a mapping scale of 1∶1,000,000 was developed by CAS (The Chinese Academy of Sciences). This database included five time periods: the late 1980s, the mid-1990s, the late 1990s, the mid-2000s and 2008. The primary data source for the land use database was Landsat MSS/TM/ETM CCD digital images. CBERS (the China-Brazil Earth Resources Satellite) and HJ-1 (small satellite constellation for environment and disaster monitoring) images were also used as a supplement for the areas uncovered by Landsat. The land-use data were classified into 25 categories, which were subsequently grouped into six classes: cropland, woodland, grassland, water body, built-up area and unused land. Detailed information on this database can be found in previous papers [22]–[26]. In this study, farmland data were extracted from 2008 land use data.

#### Meteorological Data

Meteorological data, which included the monthly maximum air temperature, minimum air temperature, precipitation, relative humidity, wind speed at 10 m height and sunshine hours from 1960 to 2010, were obtained from the national agro-meteorological stations of China maintained by Chinese Meteorological Administration (CMA) (http://cdc.cma.gov.cn). Because of the diverse terrain across China, the impact of topographic conditions on the interpolation of the meteorological data was also considered. The ANUSPLIN software [27]–[28], which was designed for spatial interpolation of climate data, was used to interpolate the meteorological data with the DEM mentioned in Section 2.1.1. These data measured monthly for the above six key factors of plant growth were then interpolated by ANUSPLIN to individual 10 km resolutions based on the digital terrain model of China (excluding Taiwan).

Before further processing, all source data were resampled into a raster dataset with a 10 km spatial resolution, and the data were transformed into the same coordinate system (Krasovsky_1940_Albers projection system, with central_meridian 105°E, double standard parallel of 27°N and 45°N, and D_Krasovsky_1940 datum).

### Method

**Figure 1:**
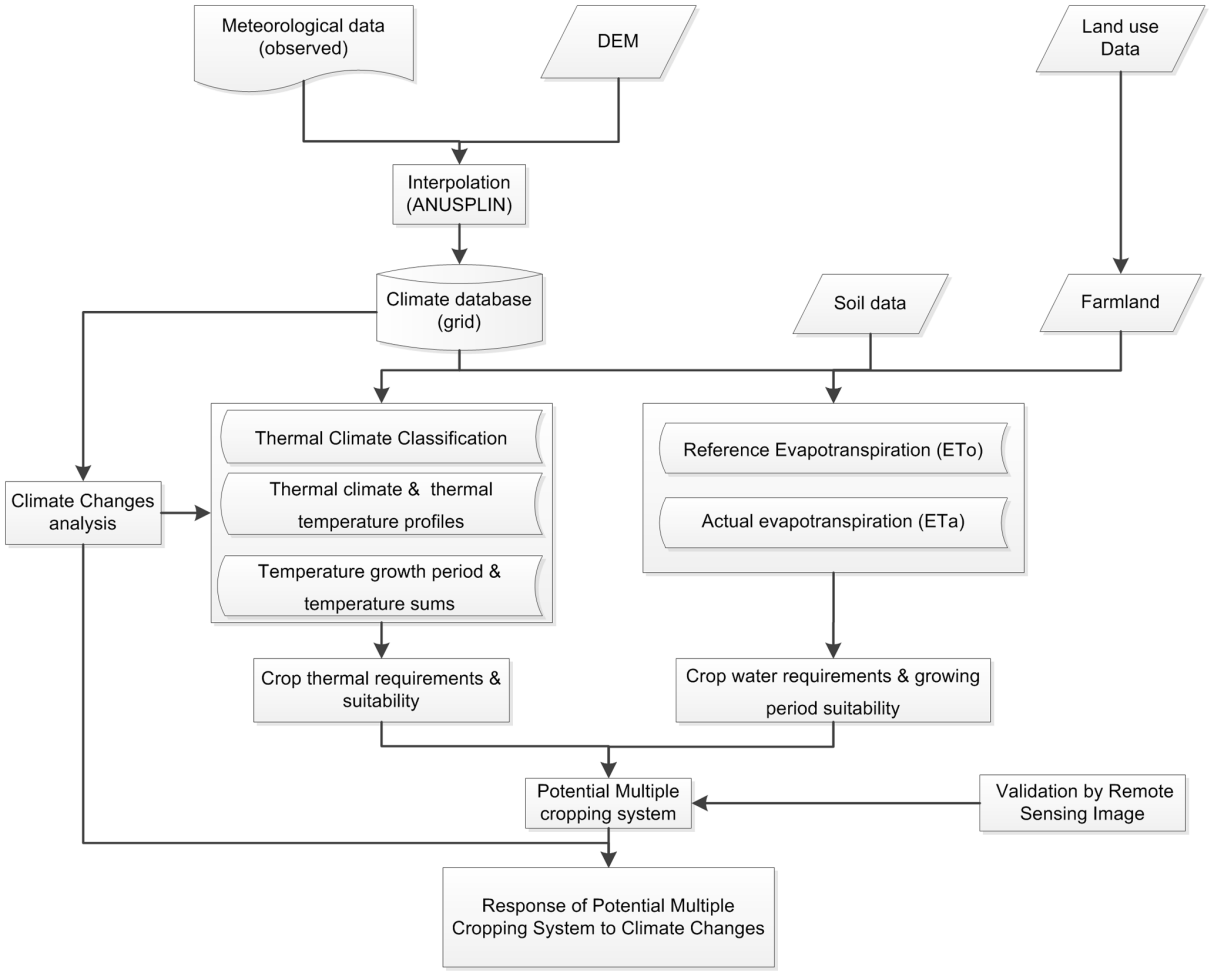
Technological framework designed for potential estimation of multiple cropping systems.

The calculation method for the potential multiple cropping systems were derived from the Global Agro-Ecological Zones Model (AEZ) which was developed in the 1970s, and updated in 2010 by the Food and Agriculture Organization (FAO) and the International Institute for Applied Systems Analysis (IIASA) [29]–[33]. The crop growth process was primarily affected by the local climate conditions during the growth period. The potential multiple cropping index (PMCI) is directly determined by the light-temperature-water condition. Figure 1 shows the technological framework designed for the calculation of the multiple cropping systems.

#### Multiple cropping zones

To assess the potential multiple cropping systems, a number of multiple cropping zones were defined by matching both the thermal and water requirements with the time required for crop growth in different latitudinal thermal climates. The following parameters were used to define the cropping zones (Table 1, Table 2): length of the growing period (LGP), number of days with mean daily temperatures above 5°C and 10°C (LGP_t = 5_ and LGP_t = 10_), accumulated temperature on days with a mean daily temperature ≥0°C and ≥10°C (TS_t = 0_ and TS_t = 10_) and accumulated temperature during the growing period with a mean daily temperature ≥5°C and 10°C (TS-G_t = 5_ and TS-G_t = 10_).

**Table 1:** Delineation of multiple cropping zones in tropical areas.

Zone	LGP	LGP_t = 5_	LGP_t = 10_	TS_t = 0_	TS_t = 10_	TS-G_t = 5_	TS-G_t = 10_
A	-	-	-	-	-	-	-
B	≥45	≥120	≥90	≥1600	≥1000	-	-
C	≥200	≥200	≥120	≥6400	n.a.	≥3200	≥2700
D	≥240	≥240	≥165	≥6400	n.a.	≥4000	≥3200
E	n.a.	n.a.	n.a.	n.a.	n.a.	n.a.	n.a.
F	≥300	≥300	≥240	≥7200	≥7000	≥5100	≥4800
G	n.a.	n.a.	n.a.	n.a.	n.a.	n.a.	n.a.
H	≥360	≥360	≥360	≥7200	≥7000	-	-

**Table 2:** Delineation of multiple cropping zones in non-tropical areas.

Zone	LGP	LGP_t = 5_	LGP_t = 10_	TS_t = 0_	TS_t = 10_	TS-G_t = 5_	TS-G_t = 10_
A	-	-	-	-	-	-	-
B	≥45	≥120	≥90	≥1600	≥1000	-	-
C	≥180	≥200	≥120	≥3600	≥3000	≥3200	≥2900
D	≥210	≥240	≥165	≥4500	≥3600	≥4000	≥3200
E	≥240	≥270	≥180	≥4800	≥4500	≥4300	≥4000
F	≥300	≥300	≥240	≥5400	≥5100	≥5100	≥4800
G	≥330	≥330	≥270	≥5700	≥5500	-	-
H	≥360	≥360	≥330	≥7200	≥7000	-	-

To reveal the impact of water conditions on the multiple cropping systems, two scenarios (irrigated and rain-fed) were considered for the calculation of the potential multiple cropping systems. The rain-fed scenarios [29] were obtained from the light-temperature-water condition, while the irrigated scenarios [30] were calculated using only the light-temperature conditions, which assumed sufficient water for crop growth. The two scenarios were consistent with two existing agricultural management methods in China (naturally rain-fed farmland and irrigated farmland). The water-limited condition referred to the LGP and TS-G mentioned previously with an additional requirement that indicated moisture was insufficient (ETa <0.4 ET_0_) [31]. According to the above criteria (Table 1, Table 2), the following eight zones were classified and mapped:

1. A. *Zone of no cropping* (too cold or too dry for rain-fed crops)
2. B. *Zone of single cropping*
3. C. *Zone of limited double cropping* (relay cropping; single wetland rice cropping may be possible)
4. D. *Zone of double cropping* (sequential cropping; double cropping with wetland rice cropping not possible)
5. E. *Zone of double cropping* (sequential cropping; wetland rice cropping possible)
6. F. *Zone of limited triple cropping* (partial relay cropping; no third cropping possible in case of two wetland rice crops)
7. G. *Zone of triple cropping* (sequential cropping of three short-cycle crops; two wetland rice crops possible)
8. H. *Zone of triple rice cropping* (sequential cropping of three wetland rice crops possible)

#### Latitudinal thermal classification

The latitudinal thermal classification was the basis for the calculation of potential multiple cropping systems (Table 3). The delineation of a latitudinal thermal regime was based on the total affected accumulation temperature above 10°C (T_sum10_), which accumulates the daily average temperatures for days in which the daily average temperature is above the appropriate threshold temperature of 10°C [34].

**Table 3:** Classification of latitudinal thermal climates.

Latitudinal thermal regime	Condition
Tropics	Tsum_10_>8000
Subtropics	4500< Tsum_10_<8000
Temperate	1500< Tsum_10_<4500
Boreal	0< Tsum_10_<1500
Arctic	Tsum_10_ = 0

#### Evapotranspiration

The reference evapotranspiration (ET_0_,mm/day) and actual evapotranspiration (ET_a_, mm/day) values were calculated using the soil-water balance calculation procedures following the methodologies outlined in “CROPWAT” [31] and “Crop Evapotranspiration” [35] for crop water requirements and growing period suitability.

The reference evapotranspiration (ET_0_, mm/day) represents the evapotranspiration from a defined reference surface which is similar to an extensive surface of green and well-watered grass of uniform height (0.12 meters) that is actively growing and completely shades the ground. The ET_0_ was calculated according to the Penman-Monteith equation [31], [36], [37].

The calculation of ET_m_ for a ‘reference crop’ is based on the assumption that sufficient water is available for uptake in the rooting zone. The value of ET_m_ is related to ET_0_ by applying the crop coefficients for the water requirements (*kc*, fractional) (Equation 1). The *kc* factors are determined by phenological development and leaf area [35]. 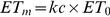
(1)


The actual uptake of water for the ‘reference’ crop was characterized by the actual evapotranspiration (ET_a_, mm/day). The calculation of ET_a_ differentiated between two possible cases depending on the availability of water for plant extraction: (i) adequate soil water availability (ET_a_ = ET_m_) and (ii) limited soil-water availability (ET_a_<ET_m_) [35].

Under adequate soil-water conditions, the value of ET_a_ was assumed to be equal to ET_m_ as long as the water balance (Wb, mm) was greater than or equal to the “readily” available soil-water (W_read_, mm). This requirement characterizes a situation in which crops are able to “easily” extract sufficient water and therefore no water stress occurs (Equation 2).
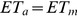
(2)when 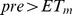 and 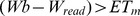


For limited water conditions, the ET_a_ can be calculated as the product of ET_m_ and the variable ρ (Equation 3).
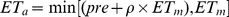
(3)


The *pre* represents the precipitation, and ρ is the quotient of the current water balance (W*b*, mm) and the readily available soil water (W*_read_*, mm). 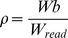
(4)


#### Soil-water balance

The volume of water available for plant uptake is calculated using a daily soil-water balance (W*b*, mm). The W*b* accounts for the accumulated daily water inflow from precipitation (pre, mm/day) or snowmelt (snm, mm/day) and the outflow from actual evapotranspiration (ETa, mm/day) and excess water loss due to runoff and deep percolation.
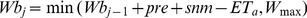
(5)


where j is the day of the year, and W_max_ (W_max_, mm) is the maximum soil water storage capacity.

## Results and Analysis

### Comparison and Verification

**Figure 2:**
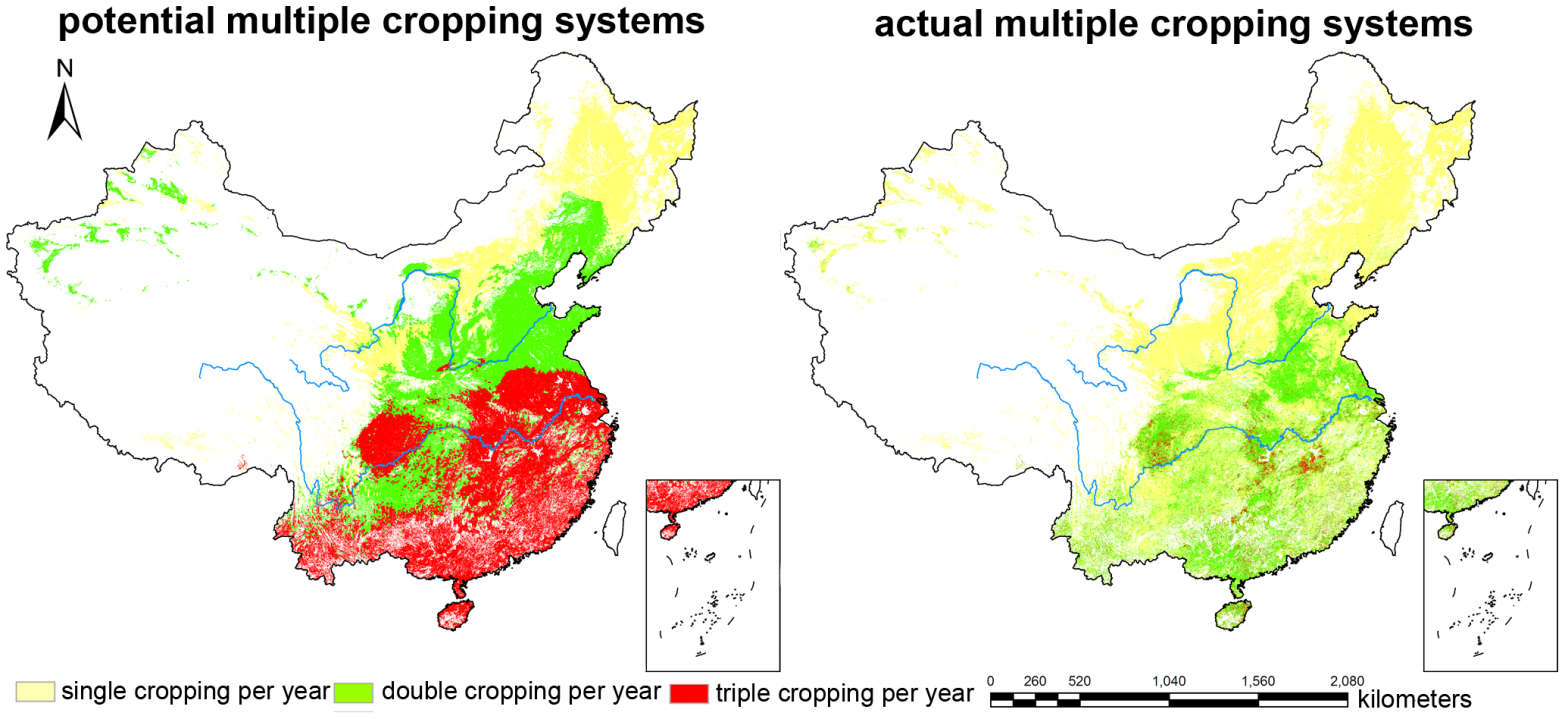
Comparison between potential and actual multiple cropping systems.

To verify the accuracy of the calculated results, the calculated potential multiple cropping system in 2000 was compared with the actual result from remote sensing monitoring based on MODIS data [11]. Three types of cropping systems were used in the actual situation. The potential multiple cropping system was also classified into three corresponding types: single cropping per year, double cropping per year, and triple cropping per year. Figure 2 demonstrates that the potential multiple cropping system was consistent with the actual system overall. For example, regions that use triple cropping (the area of 93,445 square kilometers) exist in Sichuan Basin, the Middle-Lower Yangtze Plain, the south of the Yangtze River and Hainan Island are completely simulated as triple cropping in the potential cropping system. Similarly, the single cropping in the potential cropping system (the area of 870,093 square kilometers) duplicated the actual single cropping regions. However, as shown in Table 4, the simulated MCI was larger than the actual MCI, which revealed the difference between the calculated potential multiple cropping systems and the actual systems. Farmers tended to select their multiple cropping practices following the traditional pattern which was based on previous climate conditions rather than the optimal multiple cropping systems (which are consistent with the actual local climate conditions). Moreover, many farmers preferred to grow plants that yielded greater economic profits, and the lack of young and middle-aged rural laborers who prefer to work in cities further reduced the MCI.

**Table 4:** Comparison table between potential and actual multiple cropping systems (unit: km^2^).

			actual	
		single	double	triple
	single	870,093	0	0
potential	double	981,026	301,043	0
	triple	629,786	674,971	93,445

### Spatial patterns of the potential multiple cropping system in China

**Figure 3:**
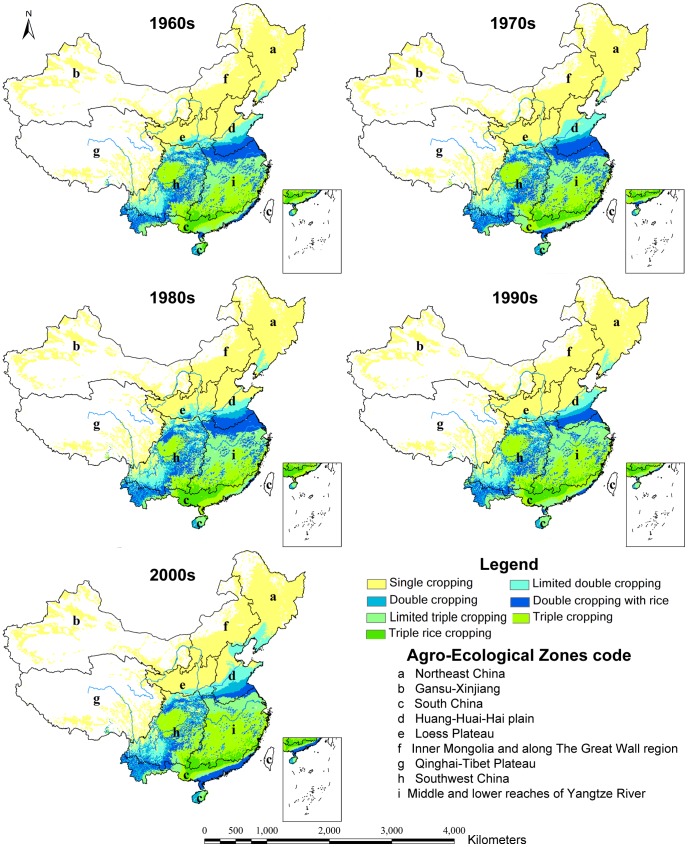
Distribution of multiple cropping systems under the rain-fed scenario.

**Figure 4:**
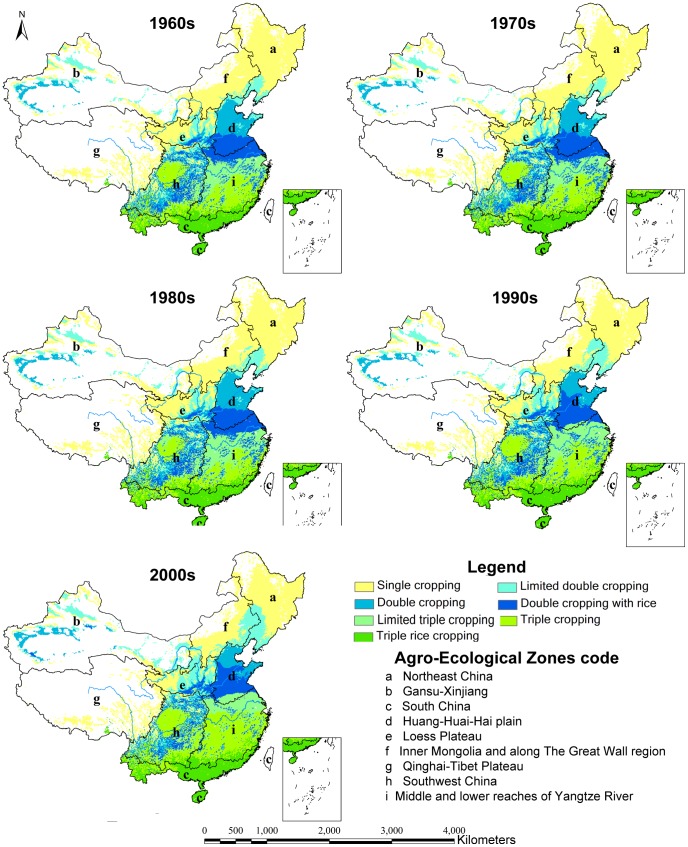
Distribution of multiple cropping systems under the irrigated scenario.

Based on the method in Section 2.2, the potential multiple cropping systems were calculated using the average climatic conditions at intervals of 10 years from the 1960s to the 2000s. Figure 3 and Figure 4 show the distribution maps of the multiple cropping systems under the rain-fed and irrigated scenarios during each 10-year period.

In the 2000s, the complexity of the potential cropping system pattern and the spatial pattern of the potential multiple cropping systems increased, as manifested from north to south and from west to east (Figure 3, Figure 4). Due to the relatively high temperature and abundant rainfall, the PMCI in the southeastern areas was high. The PMCI declined with increasing distance from the coastal line. In the northwestern portion of the country, potential multiple cropping was constrained by low temperatures and rainfall.

**Figure 5:**
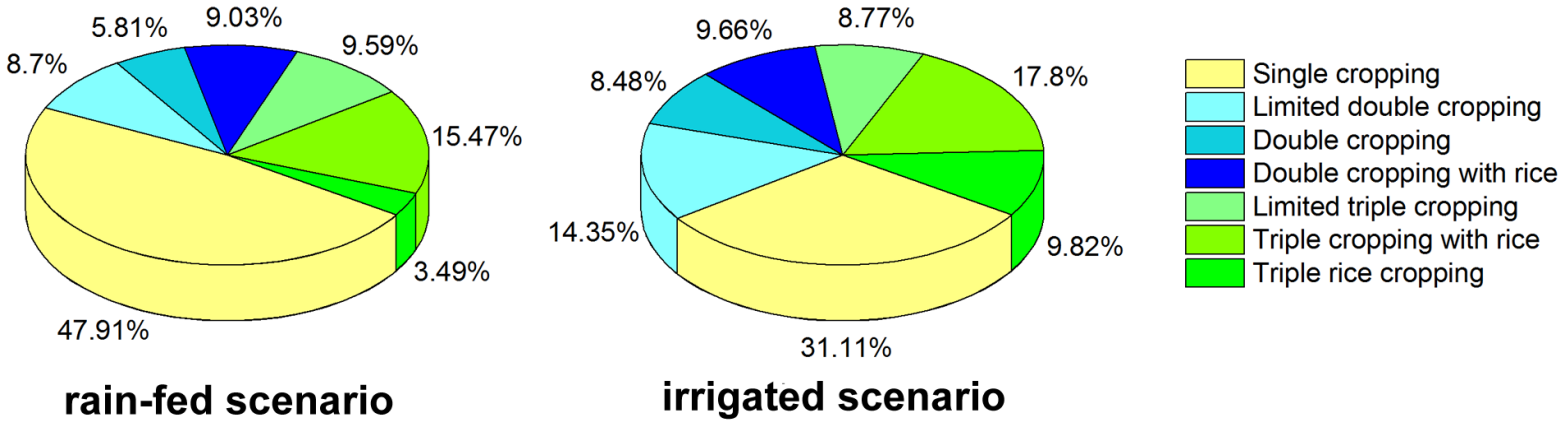
Area ratio difference of the multiple cropping systems under two scenarios in the 2000s.

Under the irrigated scenario, the triple cropping zones (including limited triple cropping, triple cropping with rice and triple rice cropping) were primarily distributed over South China and the middle and lower reaches of the Yangtze River, which cover 36.39% of the total land area of China (Figure 5). The double cropping zones (including limited double cropping, double cropping and double cropping with rice) were primarily spread across the Loess Plateau, the Huang-Huai-Hai plain and most of the Gansu-Xinjiang regions, which occupied 32.49% of the total land area of China. The single cropping zones were primarily distributed in the north of Inner Mongolia and along the Great Wall region (denoted as f) and a small portion of the Qinghai-Tibet Plateau, which occupy 31.11% of the total land area of China.

Under the rain-fed scenario, all multiple cropping zones were shifted to the south and the east, and the areas for the double and triple cropping zones were decreased by 7.84% and 8.95% respectively (Figure 5). The limited rainfall led to a decrease of the PMCI from 205% to 181%. The data showed that the irrigated condition was crucial for multiple cropping in China. Therefore, improving the construction of water facilities for farmland will benefit food productivity in China.

### Changes in the potential multiple cropping systems

The potential multiple cropping systems have undergone significant changes between the 1960s -and the 2000s. The fundamental characteristics of the changes in the potential multiple cropping systems were that the single cropping area decreased and the triple cropping area increased. Moreover, the PMCI showed an increasing trend, with the changes primarily occurring after the 1980s.

**Figure 6:**
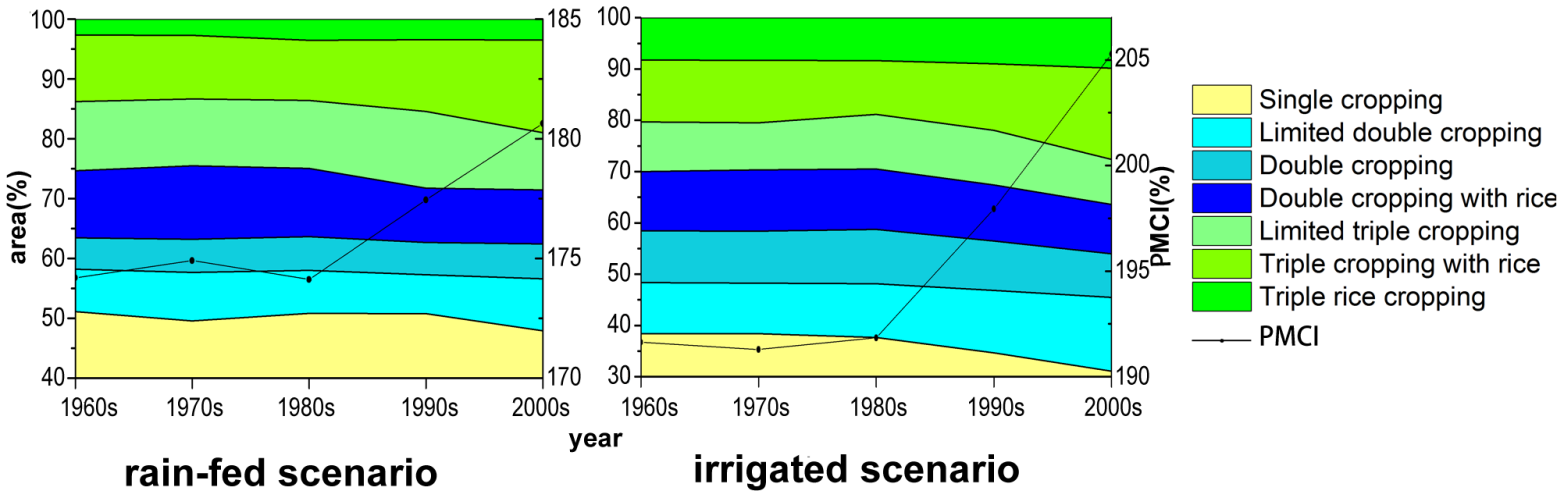
Percentage changes in the multiple cropping area and PMCI under two scenarios from the 1960s to the 2000s.

Under rain-fed scenarios from the 1960s to the 2000s, the single cropping area decreased by 3.23% (approximately 173,900 km^2^), whereas the triple cropping area increased by 3.21% (approximately 173,000 km^2^) and the PMCI increased by 7% (from 174% to 181%). No significant changes occurred from the 1960s to the 1980s, but shifts occurred in all areas and the PMCI grew rapidly from the 1980s to the 2000s. The major conversion from double cropping to triple cropping and from single cropping to double cropping took place in the time period from the 1980s to the 1990s and from the 1990s to the 2000s respectively. The size of the conversion areas was approximately equal during the two decades (Figure 6).

Under the irrigated scenarios, the single cropping area declined by 7.28% (approximately 391,600 km^2^), the triple cropping areas increased by 6.38% (approximately 343,300 km^2^), and the PMCI increased by 13% (from 192% to 205%). Major changes occurred from the 1980s to the 2000s, and the PMCI grew much more rapidly. During this period, the increase in the triple cropping area was nearly equivalent to the decrease in single cropping area, whereas the double cropping area remained unchanged as a whole(Figure 6).

### Response of potential multiple cropping systems to climate change

**Figure 7:**
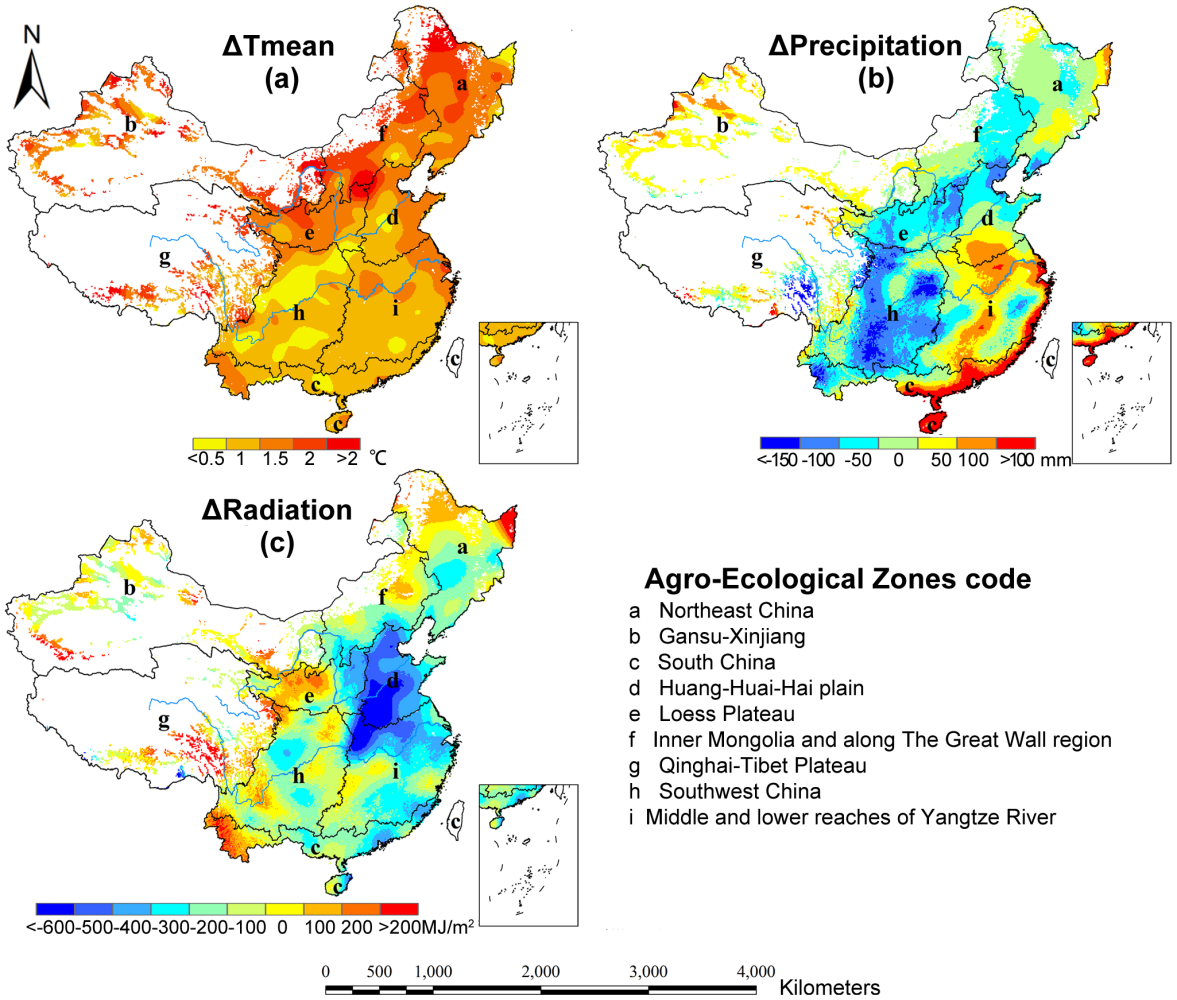
Spatial distribution pattern of climate changes from the 1960s to the 2000s in China.

**Figure 8:**
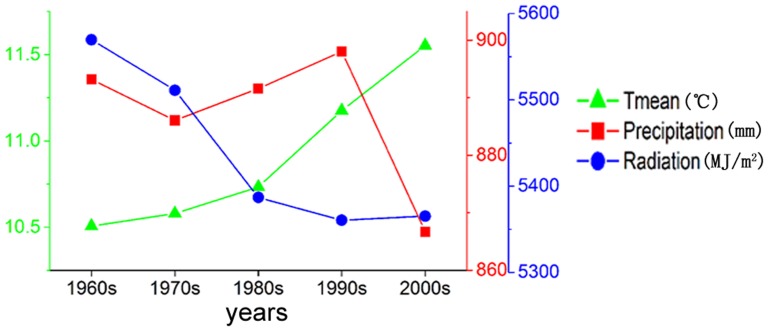
Climate changes from the 1960s to the 2000s in China.

As presented in figure 7(a, b, c), the climate change from the 1960s to the 2000s showed that the annual mean temperature, precipitation and radiation data take on different spatial change patterns. The annual mean temperature generally increased across the major cultivation regions. The line chart from the 1960s to the 2000s presented in Figure 8 reflects an obvious upward trend in the temperature change. More specifically, the temperature increased by more than 2°C in parts of the northeast, the north and the northwest of China (Figure 7a). The rate of temperature change after the 1980s (0.41°C/decade) increased more dramatically than prior to the 1980s (0.11°C/decade).

Precipitation was reduced in most regions during the entire period, especially in Southwest China, the Loess Plateau, Inner Mongolia and along the Great Wall region where the precipitation decreased by more than 10%. The areas where precipitation increased were primarily distributed in the northwest of China and the middle and lower reaches of the Yangtze River Region (Figure 7b). As an illustration, the precipitation in the Gansu-Xinjiang and Southern China region increased by 13.7% (approximately 21 mm) and 2% (approximately 31 mm), respectively. In contrast, the temporal change process showed that precipitation did not change significantly before the 1990s but took on an obvious decreasing trend (31 mm) from the 1990s to the 2000s.

Solar radiation decreased in large areas across Huang-Huai-Hai plain and most of southern China but increased in parts of Northeastern China, the Loess Plateau and the western area of Southern China (Figure 7c).The amount of radiation dropped at an average rate of 182 MJ/m^2^·decade from the 1960s to the 1980s and at an average rate of approximately 22 MJ/m^2^·decade from the 1980s to the 2010s.

**Figure 9:**
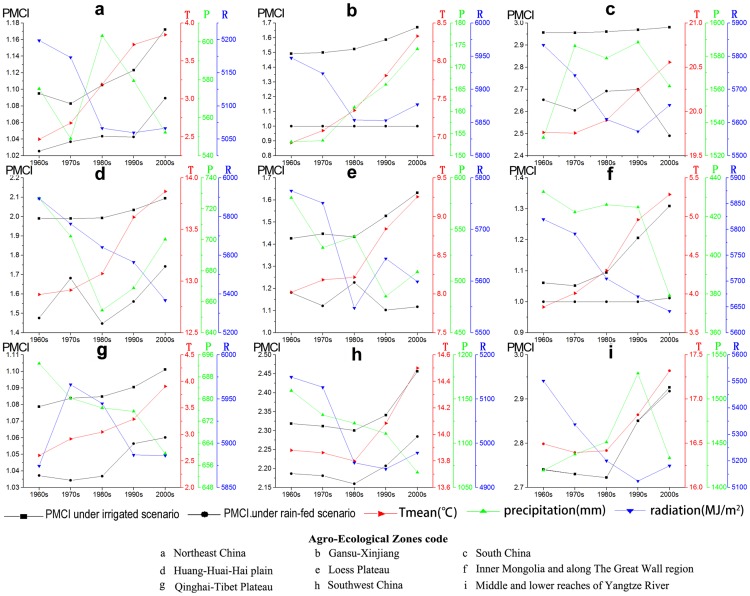
The relationship between the PMCI and climate changes in China from the 1960s to the 2000s.

The obvious spatial heterogeneity of the climate changes from the 1960s to the 2000s has an important influence on the potential multiple cropping systems in China. Temperature and precipitation were two leading factors that affected the changes of the PMCI, and Figure 8 and Figure 9 show that climate change increased the PMCI under the irrigated and rain-fed scenarios by 13% and 7% respectively in China. The increase in the PMCI was mostly due to the growth of the annual mean temperature. The gap of PMCI between rain-fed scenario and irrigated scenario was 18% in the 1960s, while this gap had increased to 24% in the 2000s.The reason for the increased gap was that the crops could not get enough water due to the reduction in rainfall, which limited the increase of the PMCI under rain-fed conditions. Consequently, in China, moisture was one of the most significant factors for the PMCI. The moisture limit will be the main factor restraining the PMCI under rain-fed scenarios in the present and perhaps also in the future.

In different regions of China, the responses of the potential multiple cropping systems to climate change took on different characteristics. In Northeast China (a), the PMCI continuously increased in two scenarios with rising annual mean temperature and declining radiation throughout the entire period. The gap in the PMCI under the two scenarios remained nearly the same, even when the precipitation sharply dropped by more than 50 mm from the 1980s to the 2000s in the rain-fed scenario. This result indicated that heat is a major factor in limiting the potential multiple cropping systems, whereas precipitation had a lesser influence.

In the Gansu-Xinjiang region (b), the potential multiple cropping systems under the rain-fed scenario was always one crop per year, whereas the PMCI under the irrigated condition rose by 0.178. This result shows that the temperature increase had a positive impact on the PMCI under the irrigated condition. The largest gap of the PMCI under the two scenarios was approximately 0.7. However, a small increase in precipitation did not cause the increase in the PMCI under the rain-fed condition due to the extreme deficiency of rainfall.

In Southern China (c), the PMCI under the irrigated scenario was close to 3, which is primarily due to the effect of abundant heat on crop growth. The PMCI under the rain-fed scenario took on an obvious decreasing trend, and the PMCI gap under the two scenarios fluctuated between 0.26 and 0.5. This result was primarily due to the highly uneven rainfall.

In Huang-Huai-Hai plain (d), the annual mean temperature increased by 1.5°C from the 1960s to the 2000s, which led to an increase in the PMCI of 0.103 under the irrigated scenario. Under the rain-fed scenarios, the PMCI increased by 0.266, and displayed a fluctuation that first increased, subsequently decreased, and finally increased again. The change in the PMCI was primarily due to the sharp decrease in radiation by more than 500 MJ/m^2^·year and the increase in precipitation during certain periods. The gap between the two scenarios was approximately 0.35, which indicates that water restricts crop growth in this region to a certain extent.

In the Loess Plateau (e), Inner Mongolia and along the Great Wall region (f), the annual mean temperature and PMCI under irrigated conditions showed a simultaneous growth trend, whereas the PMCI under the rain-fed condition changed little, resulting in larger gaps. A potential reason for this finding was that the declining precipitation caused a moisture shortage for the crops.

The PMCI of the Qinghai-Tibet Plateau (h) under the two scenarios were both lower than those of other regions, which rose slightly with increasing temperatures, and the gaps were rarely affected by a reduction in precipitation. The main reason for this appeared to be that the temperature was too low (below 5°C) for normal crop growth.

In Southwest China (i) and the middle and lower reaches of the Yangtze River (j), the annual mean temperature and PMCI under the two scenarios showed a concurrent growth trend. Although the precipitation fluctuated, the PMCI gap under the two scenarios was almost unchanged. This result shows that these areas received sufficient rain for plant growth under the prevailing heat condition, which implies that these areas are the most suitable zones for plant growth in China.

## Conclusions and Discussions

The multiple cropping systems demonstrate the efficient use of water, soil, light energy, and other natural resources [38], all of which are important for agricultural production and food security. Wherever the natural conditions allow, it is necessary to adopt high intensity cultivation to secure efficient food supply in China [2].

This study focused on the calculation and analysis of potential multiple cropping systems in China based on multi-source data. We also analyzed the changes in the potential multiple cropping systems in response to climate change in China from the 1960s to the 2010s. Conclusions from this work are as follows [39]–[42]:

(1) The spatial pattern of potential multiple cropping systems in China displays tremendous heterogeneity, and an increasing complexity from northern China to southern China, and from western China to eastern China. The decrease in the single cropping area, and the increase in the triple cropping area, are the fundamental characteristics of the potential multiple cropping systems change from the 1960s to the 2000s. The rate of increase of PMCI after the 1980s was far larger than that before the 1980s. During the studied period, the PMCI gap between rain-fed and irrigated scenarios increased from 18% to 24%, which indicated noticeable growth of water supply limitations under the rain-fed scenario. Irrigation conditions were important for multiple cropping practices, underlining the importance of the construction of water facilities would be beneficial for food production in China.

(2) The obvious spatial heterogeneity of climate change from the 1960s to the 2000s had a significant influence on the potential multiple cropping systems in China. The climate change caused the PMCI under the irrigated and rain-fed scenarios to increase by 13% and 7% respectively across the whole of China. Furthermore, the growth of the annual mean temperature was identified as the main reason for the increase of the PMCI.

(3) In the different regions of China, the response of the potential multiple cropping systems to climate change took on different characteristics. In Southern China, the PMCI under the rain-fed scenario took on an obvious decreasing trend because of highly uneven rainfall, although precipitation was abundant. In the Northeast and Qinghai-Tibet Plateau, the heat severely restricted crop growth, and in Gansu-Xinjiang region, Loess Plateau, Inner Mongolia and along the Great Wall region, precipitation was the major factor that limited the potential multiple cropping systems. In Southwest China and the middle and lower reaches of the Yangtze River, the PMCI showed a simultaneous growth trend under both the irrigation and rain-fed scenarios, although precipitation fluctuated.

This study calculated the potential multiple cropping systems based on meteorological data, and subsequently analyzed the relationships between the potential multiple cropping systems and climate change, which assist in dealing with the issue of food security in China. However, certain limitations exist in this research. For instance, (1) due to the lack of irrigation data, we were not able to simulate the actual multiple cropping systems according to real irrigation conditions, instead we simulated two different scenarios (rain-fed and irrigated scenarios); (2) it was assumed that the irrigated scenarios would supply sufficient water for crop growth, but in practice the availability of water under the irrigation scenario still poses limitations to crop growth; (3) extreme weather conditions (i.e., freezing or high temperatures, rainstorms and heavy snow) may have an extreme effect on the multiple cropping systems, but these factors were not taken into consideration in our research.
